# Tumor-derived exosomes promote the in vitro osteotropism of melanoma cells by activating the SDF-1/CXCR4/CXCR7 axis

**DOI:** 10.1186/s12967-019-1982-4

**Published:** 2019-07-19

**Authors:** Francesco Mannavola, Marco Tucci, Claudia Felici, Anna Passarelli, Stella D’Oronzo, Francesco Silvestris

**Affiliations:** Department of Biomedical Sciences and Human Oncology, University of Bari ‘Aldo Moro’, P.za Giulio Cesare, 11-70124 Bari, Italy

**Keywords:** Melanoma, Exosomes, Bone metastasis, CXCR7, CXCR4

## Abstract

**Background:**

Bone metastases occur rarely in patients suffering from malignant melanoma, although their onset severely worsens both prognosis and quality of life. Extracellular vesicles (EVs) including exosomes (Exos) are active players in melanoma progression involved in the formation of the pre-metastatic niche.

**Methods:**

Trans-well assays explored the basal migratory and invasive potential of four melanoma cell lines and investigated their different propensity to be attracted toward the bone. Exosomes were purified from cell supernatants by ultracentrifugation and explored in their ability to influence the bone tropism of melanoma cells. The molecular machinery activated during this process was investigated by RT-PCR, droplet digital-PCR, flow-cytometry and Western blot, while loss of function studies with dedicated siRNAs defined the single contribute of CXCR4 and CXCR7 molecules.

**Results:**

Melanoma cells revealed a variable propensity to be attracted toward bone fragments. Gene profiling of both osteotropic and not-osteotropic cells did not show a different expression of those genes notoriously correlated to chemotaxis and bone metastasis. However, bone conditioned medium significantly increased *CXCR4*, *CXCR7* and *PTHrP* expression solely to osteotropic cells, while their Exos were able to revert the original poor bone tropism of not-osteotropic cells through *CXCR7* up-regulation. Silencing experiments also demonstrated that membrane expression of CXCR7 is required by melanoma cells to promote their chemotaxis toward SDF-1 gradients.

**Conclusions:**

Our data correlated the osteotropism of melanoma cells to the activation of the SDF-1/CXCR4/CXCR7 axis following the exposition of tumor cells to bone-derived soluble factors. Also, we demonstrated in vitro that tumor-derived Exos can reprogram the innate osteotropism of melanoma cells by up-regulating membrane CXCR7. These results may have a potential translation to future identification of druggable targets for the treatment of skeletal metastases from malignant melanoma.

**Electronic supplementary material:**

The online version of this article (10.1186/s12967-019-1982-4) contains supplementary material, which is available to authorized users.

## Background

Bone metastases occur approximately in 15% of patients with cutaneous melanoma leading to typical skeletal-related events (SRE) that worsen prognosis and quality of life [1] and although recent strategies improved the morbidity and delayed the SRE development, the survival of these patients is severely affected [2].

Metastases of the skeleton arise from a stepwise process that induces cancer cells to acquire an invasive behaviour, including their detachment from primary tumor, spreading throughout the blood stream, housing within the bone niche and growing [3]. Previous studies demonstrated that pro-angiogenic cytokines, growth factors and extracellular vesicles (EVs) including exosomes (Exos) are released by melanoma cells to prepare a favourable soil for their outgrowth at pre-determined sites [4]. To this, the transforming growth factor-β (TGF-β), the parathyroid hormone-related peptide (PTHrP), the receptor activator of nuclear factor kappa-B ligand (RANKL) as well as the interleukin (IL)-6 have been proven to be variably enrolled in favouring the tumor cell seeding within the pre-metastatic niche as well as metastasis development [5–8]. Additionally, other studies [9] emphasize the role of the bone microenvironment in which the stromal cell-derived factor (SDF)-1, a chemoattractant released by mesenchymal cells, recruits circulating cancer cells, particularly from epithelial tumors like breast and prostate cancers, bearing CXCR4 (C-X-C chemokine receptor type 4).

Exosomes are endosomal-derived nanovesicles produced by normal and malignant cells involved in the intercellular communications whose efficiency depends on their molecular cargos of soluble factors, proteins and nucleic acids [10]. Exosomes are increased in sera of patients with melanoma and have recently emerged as active players of tumor progression in relation to their involvement in the metastatic machinery by activating the epithelial-to-mesenchymal transition (EMT), favouring the immune evasion and driving the formation of the pre-metastatic niche [11–13]. Notwithstanding recent data support the involvement of tumor-derived Exos in the metastatic colonization of the skeleton in lung cancer [14], their contribution in influencing a similar behaviour in melanoma cells remains indeed poorly investigated.

Here, we explored the potential role of tumor-derived Exos in influencing the migration and invasiveness of melanoma cells toward the bone. In parallel, we attempted to address the molecular mechanisms required for the activation of these functional properties by focusing on the SDF-1/CXCR4/CXCR7 signaling.

## Methods

### Cell lines and bone specimens

Melanoma (SK-Mel28, WM266, LCP and LCM) and breast cancer (MDA-MB231) cell lines (ATCC, Rockville, MD, USA) were cultured in Exo-free complete medium. Overnight starvation with FBS-depleted medium was used to synchronize the cell cycle before each experiment. Scraps of cancellous bones were recovered from 5 healthy subjects suffering of post-traumatic orthopaedic surgery and used to generate small bone fragments of 3–5 mm^3^, or cultured for 24 h with free-culture medium to achieve bone conditioned medium (BCM) [8].

### Exosome purification and characterization

Exosomes were purified by ultracentrifugation of supernatants from 48-h cultured melanoma cells [15]. Briefly, dead cells, debris, protein aggregates and microvesicles were removed by both centrifugation and mechanical filtration using Millipore filter of 0.22 µm diameter. Supernatants were then twice centrifuged at 100,000×*g* for 70 min at 4 °C to obtain Exos that were stored at − 80 °C in PBS aliquots of 100 µl. A limited number of samples were randomly selected to verify the size distribution and concentration of vesicles by using the NanoSight NS300 instrument (Malvern Instruments, Malvern, UK), while the transmission electron microscopy (TEM) defined the morphology of vesicles. After the measurement of protein amount using the Bradford protein assay (Bio-Rad), Exo preparations from each sample were verified by measuring the expression of CD63, CD81 (eBioscence) and CD9 (BD Pharmigen) by flow-cytometry [16] with dedicated mouse anti-human monoclonal antibodies (MoAbs). For this purpose, 30 µg of Exos were previously conjugated with 4 µm diameter aldehyde/sulfate latex beads (Invitrogen, Carlsbad, CA) [17], while mouse IgG1 was the isotypic control. Moreover, to further validate the purity of Exo preparations, western blots (WB) were performed to measure the levels of CD81, TSG101, calnexin (CANX) and bovin serum albumin (BSA) in accordance to Minimal Information for Studies of Extracellular Vesicles (MISEV) guidelines [18].

The ability of melanoma cells to incorporate Exos was also investigated by confocal microscopy (Nikon Instr., Lewisville, TX). Briefly, 1 × 10^4^ melanoma cells were cultured for 4 h with 50 µg/ml of Exos previously bound to a red lipophilic fluorescent dye (PKH26; Sigma-Aldrich, St Louis, MO, USA) [14]. Then, cells were stained with FITC-conjugated phalloidin (Invitrogen), while nuclei counterstained with DAPI (4′,6-diamidino-2-phenylindole; Sigma Aldrich).

### Migration and invasion assay

Trans-well plates of 8 µm diameter (Corning Incorporated, NY) were used to investigate the migratory behaviour of melanoma cells, while invasiveness was assessed by the BioCoat Matrigel cell culture chambers (Becton–Dickinson Bioscience, MA). MDA-MB231 cells were the positive control in relation to their metastatic bone tropism [19]. For both migration and invasion assays, 1 × 10^4^ cells were seeded onto the upper chamber in presence of RPMI supplemented with 1% FBS. The lower chamber was filled with 10% FBS or bone fragment as chemoattractant, while 1% FBS was the negative control. Then, cells adherent on the upper surface of the membrane were removed at different time points (24 and 48 h), while those trapped in the underside of the insert were fixed with 4% paraformaldehyde, stained with DAPI and visualised under a UV microscope (Leica, Heidelberg, Germany). DAPI^+^ cells were counted in ten random fields of 0.2 mm^2^ at 40× magnification.

Further experiments explored the Exo role in influencing the osteotropic attitude of melanoma cells. To this, both migration and invasion assays were completed as previously described although the lower chamber was filled with Exos (50 µg/ml) derived from autologous (a-Exos) or heterologous melanoma cells (h-Exos). Each experiment was completed in triplicate.

### Gene expression analyses

The basal expression of 27 genes mainly implicated in cell migration and bone tropism were investigated by real-time (RT)-PCR in unstimulated melanoma cells using a 96-well custom plate (BioRad). GAPDH was the housekeeping gene to calculate the 2^−Δct^. In addition, the potential effect on gene expression levels after 6-h of melanoma cell stimulation by BCM, Exos or both, was explored by QX200 droplet digital (dd)-PCR. Data were analyzed by the QuantaSoft software (BioRad). Genes and relative primers used for RNA amplification are listed in Additional file 1: Table S1. Experiments were completed in biological triplicate and results were expressed in terms of fold change using *GAPDH* as the housekeeping gene.

### CXCR4 and CXCR7 expression analyses

Based on the results of dd-PCR, membrane and intracellular expression of CXCR4 and CXCR7 by melanoma cells were investigated by flow cytometry using dedicated mouse anti-human MoAbs (Abcam), while the mean fluorescence intensity (MFI) ratio was calculated with respect to IgG1 isotypic control. Both membrane and cytoplasmic protein fractions from melanoma cells were obtained using the Mem-PERM Plus Kit (Thermo Scientific) and the relative CXCR4 and CXCR7 levels measured by WB. Either pan-cadherin or alpha-tubulin (Abcam) were used as intra-assay control for membrane and intracellular measurements, respectively, as described [20]. Protein expression was calculated in terms of optical density (O.D.) by ImageQuantTL (GE Healthcare, UK), while differences between stimulated (BCM or Exos) and unstimulated cells were expressed in terms of ratio [21]. Additionally, CXCR4 and CXCR7 levels on melanoma-derived Exos were measured by flow-cytometry as previously described.

### Loss of function study

Further experiments assessed the contribute of either CXCR4 or CXCR7 in conditioning the osteotropism of melanoma cells. Therefore, their expression was restrained by small interfering RNAs (siRNAs) using the following primers: 5′-GGCAGUCCAUGUCAUCUACTT-3′ and 5′-GUAGAUGACAUGGACUGCCTT-3′ for CXCR4; 5′-GGAUGACACUAAUUGUUAGTT-3′ and 5′-CUAACAAUUAGUGUCAUCCTT-3′ for CXCR7 (Life Technologies, CA, USA). Briefly, 1 × 10^6^ LCP and SK-Mel28 cells were treated for 48 h with 3.75 µl/ml of Lipofectamine 3000 Reagent (Invitrogen, CA, USA) to induce transient transfection in presence of anti-CXCR4 (30 nmol/l) or anti-CXCR7 (60 nmol/l) siRNAs. Cells treated with 3.75 µl/ml of Lipofectamine 3000 reagent or scramble probes (Ambion) were the controls.

Since both CXCR4 and CXCR7 share the SDF-1 ligand, silenced melanoma cells were explored in their propensity to migrate and invade toward human-recombinant SDF-1 (R&D Systems, MN, USA) either in presence or absence of Exos. The optimal concentration of SDF-1 used in these experiments, namely 100 ng/ml, was similar to that detected in BCM by ELISA (96.4 ± 17.2 ng/ml).

### Statistical analysis

Data were analysed by the Mann–Whitney test for not-parametric distributions, while One-way ANOVA was used to analyse the differences between basal levels of CXCR4 and CXCR7 by melanoma cells. Data were was considered statistically significant with a p < 0.05.

## Results

### Melanoma cells differently migrate toward the bone

**Fig. 1:**
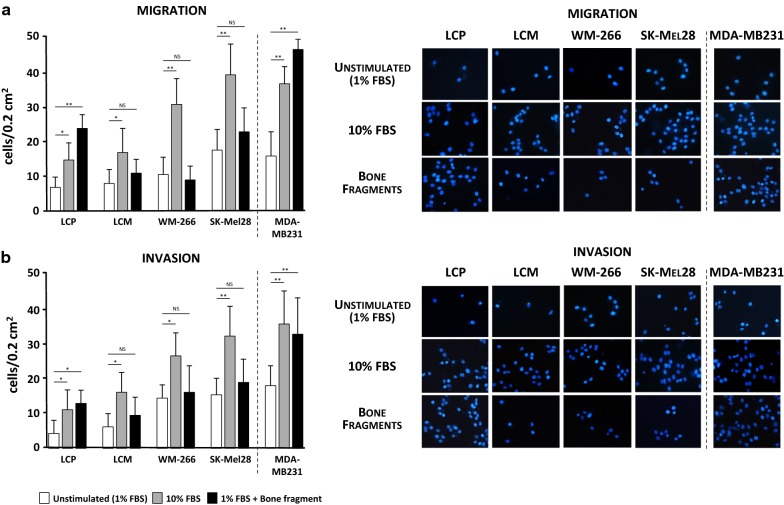
Migration and invasion assays of melanoma cell lines. **a** The migration of melanoma cells was enhanced by 10% FBS as compared to unstimulated cells (average: 25.3 ± 5.5 vs 11.4 ± 2.8 cells/0.2 cm^2^). The stimulation with bone fragment significantly increased the migration of LCP with respect to unstimulated cells, while produced modest effects on LCM, WM-266 and SK-Mel28. MDA-MB231 breast cancer cells were the positive control. **b** The invasion assay also confirmed the general invasive attitude under the FBS stimulation (WM-266: 26.3 ± 7.8 cells/0.2 cm^2^; SK-Mel28: 38.9 ± 9.7 cells/0.2 cm^2^), although only LCP showed a significant increase of invasiveness (13.1 ± 3.4 vs 4.4 ± 2.9 cells/0.2 cm^2^) when stimulated with bone fragment as for MDA-MB231 cells (33.6 ± 10 vs 18.7 ± 4.8 cells/0.2 cm^2^). Bars are mean ± SEM. NS: not significant; *p < 0.05; **p < 0.01. Images on the right side are representative of fluorescence microscopy at ×40 magnification of cells trapped within the trans-well membranes as effect of different stimulations

The first set of experiments evaluated the migration tendency of melanoma cell lines. As shown in Fig. 1a, c, melanoma cells stimulated for 24-h with 10% FBS increased their basic migratory propensity with respect to unstimulated cells (p < 0.01 in all instances) and this occurred in a fashion almost similar to MDA-MB231. Both SK-Mel28 and WM-266 cells showed the highest migratory potential (39.6 ± 9.0 and 31 ± 7.5 cells/0.2 mm^2^) as compared to LCP and LCM (14.8 ± 1.9 and 17 ± 6.8 cells/0.2 mm^2^, respectively). However, the migratory capacity of LCP cells also increased once exposed to the stimuli of bone fragments (24.3 ± 4.4 cells/0.2 mm^2^; p < 0.01), as for MDA-MB231 cells (46.2 ± 3.6 cells/0.2 mm^2^; p < 0.01). By contrast, bone fragments exerted only a modest effect on LCM, WM-266 and SK-Mel28. Similar results were obtained by measuring the invasiveness in the same melanoma populations (Fig. 1b, c), as well as in cells stimulated for 48-h (data not shown). In relation to these preliminary findings and for the next experiments, LCP cells were arbitrarily defined as ‘osteotropic’, whereas WM-266 and SK-Mel28 as ‘not-osteotropic’. Moreover, 24-h stimulation was arbitrarily defined as the optimal time point for the next trans-well assays.

### Exosomes influence the migratory behaviour of melanoma cells

**Fig. 2:**
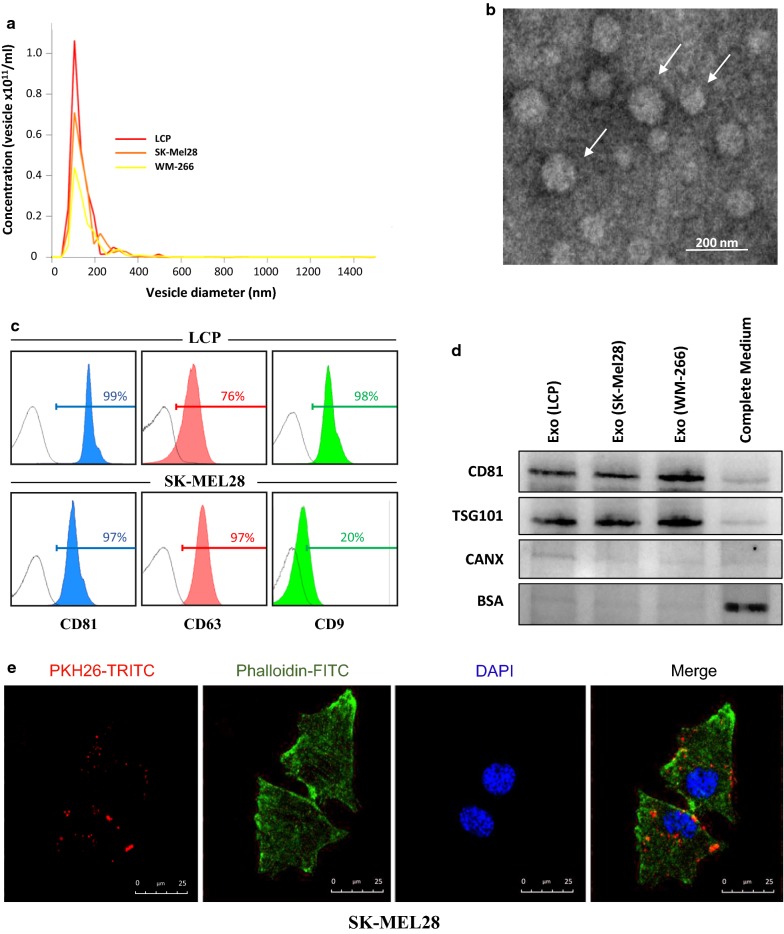
Characterization of Exos from melanoma cells. **a** Exosome preparations were analyzed using NanoSight technology. Histogram represents the size distribution of nanovesicles purified from LCP, SK-Mel28 and WM-266 conditioned supernatants, and the analysis reveals more than 80% of vesicles with a diameter ranging from 30 to 150 nm. Results are the mean from three different measurements. **b** Representative panel showing Exos preparations by TEM reveals the presence of nanovesicles with typical cup-shaped morphology (arrows). **c** Flow-cytometry described the CD81, CD63 and CD9 tetraspanin expression in Exo preparations from representative LCP and SK-Mel28 cells whose values were higher than 95%. **d** Western blots for Exo markers (CD81 and TSG101) and potential contaminants (CANX, BSA) of Exo preparations isolated from supernatants of melanoma cells. Fresh Exo-free complete medium was used as control. **e** Representative confocal microscopy images at ×40 magnification showing the up-take of PKH26-labeled Exos (50 µg/ml) by melanoma cells after 4-h. Large Exo clusters (red dots) were found to be mostly distributed in the cytoplasm of SK-Mel28 cells (merge), whose actin filaments and nuclei were stained with phalloidin (green) and DAPI (blue), respectively

Next, we investigated the potential influence of Exos on both migration and invasiveness of melanoma cells. Exosomes were purified from supernatants of WM-266, SK-Mel28 and LCP melanoma cells at a mean concentration of 1.6 × 10^10^ vesicles/ml, 1.1 × 10^11^ vesicles/ml and 1.9 × 10^11^ vesicles/ml, respectively. Figure 2 illustrates specific characteristics of Exos, including the 30–150 nm diameter revealed in more than 80% of vesicles (panel a), the typical cup-shaped morphology by TEM (panel b), and the presence of CD81, CD63 or CD9 tetraspanins (panel c). Western blot analyses (Fig. 2d) demonstrated that Exo preparations were positive for typical markers of EVs (CD81 and TSG101), while excluded the possible contamination by large-EVs (> 200 nm) or non-EV structures, such as protein aggregates, since all the samples resulted negative for both CANX and BSA as compared to complete control medium. Finally, Fig. 2e evidences the uptake of red-fluorescent labeled Exos by SK-Mel28 cells whose cytoplasm was engulfed of clusters formed by h-Exo from LCP.

**Fig. 3:**
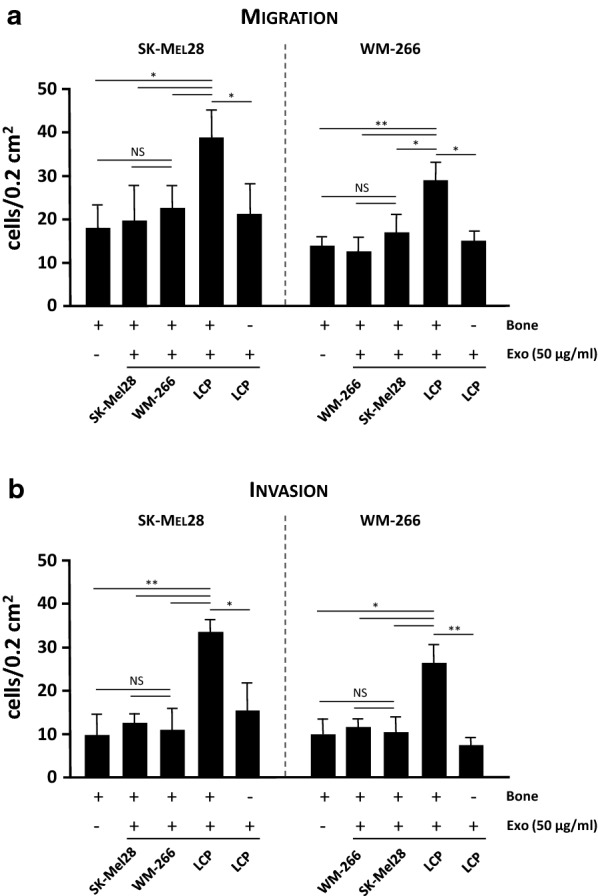
Exosomes influence the osteotropism of melanoma cells. The effects of Exos (50 µg/ml) on bone tropism of not-osteotropic SK-Mel28 and WM-266 cells were explored using bone fragments as chemoattractant. Both migration (**a**) and invasion (**b**) were significantly enhanced by h-Exos from osteotropic LCP, while they were not influenced by either a-Exos or h-Exos from not-osteotropic cells. The effect of LCP-derived Exos was abrogated in absence of bone fragments, thus suggesting their ability to sensitize not-osteotropic cells to the bone chemoattraction rather than stimulate their migration. Bars are mean ± SEM. *NS* not-significant; *p < 0.05; **p < 0.01

We then explored the effect of stimulation with both Exos and bone fragments on the migration and invasiveness of not-osteotropic cells, namely SK-Mel28 and WM-266. As shown in Fig. 3a, both a-Exos and h-Exos from non-osteotropic cells poorly improved the migration of SK-Mel28 (19.5 ± 7.8 and 23.2 ± 4.1 cells/0.2 mm^2^, respectively) and WM-266 cells (10.8 ± 3.2 and 16.2 ± 4.8 cells/0.2 mm^2^) as compared to controls (SK-Mel28: 18.2 ± 4.8 cells/0.2 mm^2^; WM-266: 13.3 ± 2.4 cells/0.2 mm^2^; p = ns in both instances). By contrast, the migration was significantly increased in the presence of h-Exos from osteotropic LCP (SK-Mel28: 38.4 ± 5.3 cells/0.2 mm^2^; WM-266: 27.1 ± 5.2 cells/0.2 mm^2^; p < 0.05), although this effect was abrogated by the removal of bone fragment (SK-Mel28: 20.9 ± 6.1 cells/0.2 mm^2^; WM-266: 15.5 ± 2.6 cells/0.2 mm^2^). Similar results occurred by exploring the invasive behaviour of SK-Mel28 and WM-266 (Fig. 3b). Taken together, these data suggested a potential role of Exos from melanoma cells in conferring osteotropic function in heterologous cells.

### Basal gene expression profile of melanoma cells does not correlate with osteotropic propensity

To identify possible deregulated genes reflecting the osteotropic propensity of melanoma cells, we compared the transcriptional profiles of osteotropic LCP to not-osteotropic SK-Mel28 and WM-266 cells. Therefore, gene expression analyses investigated the expression of several genes potentially implicated in cell migration and chemotaxis.

**Fig. 4:**
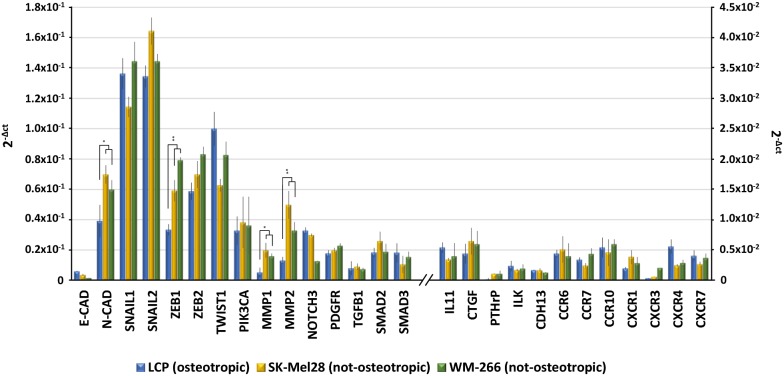
Basal gene expression of ‘osteotropic’ and ‘not-osteotropic’ melanoma cells. Real time-PCR analyzed osteotropic (LCP) and not-osteotropic cells (SK-Mel28 and WM-266) in their basal expression of 27 genes involved in the epithelial-to-mesenchymal transition (EMT), chemotaxis and bone metastasis development. Both osteotropic and not-osteotropic cells exhibit an EMT profile as evidenced by the over-expression of *N*-*Cadherin* (*N*-*CAD*), *SNAIL*, *ZEB*, *TWIST* and *MMP*. By contrast, genes notoriously described to be implicated in chemotaxis and bone metastasis were apparently down-regulated at baseline in each cell line. Results are expressed as 2^−Δct^ and bars represent mean ± SEM. *p < 0.05; **p < 0.01

As showed in Fig. 4, genes regulating the EMT-machinery, including *N*-*Cadherin* (*N*-*CAD*), *SNAIL, ZEB* and *TWIST1,* were found variably up-regulated in both osteotropic and not-osteotropic cells with the exclusion of *E*-*CAD.* The mRNA basal levels of *N*-*CAD* (p < 0.05), *ZEB1* (p < 0.001), *MMP1* (p < 0.05) and *MMP2* (p < 0.001) were significantly increased in not-osteotropic cells with respect to osteotropic LCP. However, the other genes resulted poorly expressed in explored cell lines.

These findings demonstrate that both osteotropic and not-osteotropic cells showed at baseline a similar EMT signature, poorly correlated with the bone tropism.

### Exosomes from osteotropic cells induce CXCR7 up-regulation in not-osteotropic melanoma cells

**Fig. 5:**
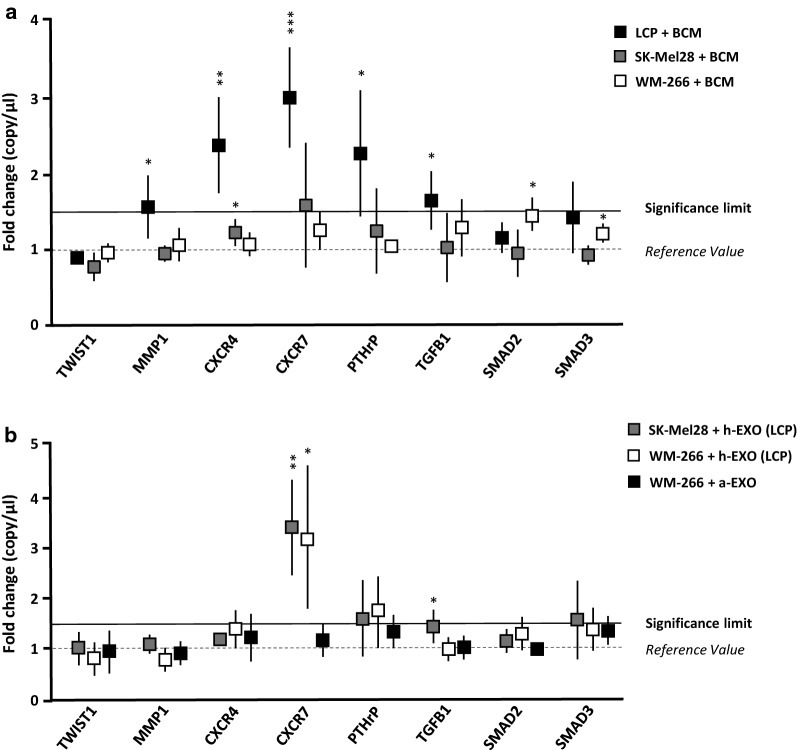
The exposition of melanoma cells to BCM or h-Exos induces transcriptomic modifications. **a** LCP, SK-Mel28 and WM-266 cells were analyzed by dd-PCR to identify potential gene expression variations induced by BCM stimulation. Dot plots refer to representative genes whose levels were influenced by BCM. Levels of *MMP*-*1*, *CXCR4*, *CXCR7*, *PTH*-*rP* and *TGF*-*β* were significantly up-regulated in LCP, while remained unchanged in SK-Mel28 and WM-266 cells. **b** Similar analyses explored the effects of LCP-derived Exos on not-osteotropic SK-Mel28 and WM-266 cells. A significant up-regulation of *CXCR7* (> 3-fold increase) occurred in both cell lines, while stimulation of WM-266 cells with a-Exos failed to produce significant difference from baseline values. Results are expressed as fold change from basal mRNA concentration (copy/µl) and represent mean ± SEM. A threshold > 1.5-fold change (bold line) was arbitrarily identified as significant. *p < 0.05; **p < 0.01; ***p < 0.001

Since the baseline EMT gene expression profile was not correlated with the osteotropic behaviour of LCP, SK-Mel28 and WM-266, we hypothesized that a major effect may occur within the bone microenvironment. To verify this, we measured the expression of the same genes following the stimulation of melanoma cells with BCM. As shown in Fig. 5a, osteotropic LCP underwent a significant up-regulation from baseline of *CXCR7* (2.9 ± 0.6-fold increase; p < 0.001), *CXCR4* (2.3 ± 0.5-fold increase; p < 0.01) and *PTH*-*rP* (2.2 ± 0.7-fold increase; p < 0.05), while *MMP1* and *TGF*-*β1* were slightly increased (≈ 1.5-fold increase; p < 0.05). By contrast, the transcriptional profile of not-osteotropic SK-Mel28 and WM-266 cells remained almost unchanged following the BCM stimulation, except for a minimal increment of *CXCR7* (≈ 1.5-fold increase; p = ns) for SK-Mel28. Similar experiments explored the effects induced by h-Exos from osteotropic LCP on SK-Mel28 and WM-266 cells (Fig. 5b). As shown, the stimulation with h-Exos dramatically increased the expression of *CXCR7*, as compared to unstimulated cells, both in SK-Mel28 (3.3 ± 0.8-fold increase; p < 0.05) and WM-266 cells (3.1 ± 1.3-fold increase; p < 0.01). On the contrary, no transcriptional modifications were observed in not-osteotropic WM-266 cells stimulated with a-Exos, thus suggesting that *CXCR7* up-regulation was putatively responsible of the osteotropic behavioral changes induced by LCP-derived Exos. To validate these results, we further investigated the effects of BCM or h-Exos stimulation on protein levels of both CXCR4 and CXCR7 in melanoma cells.

**Fig. 6:**
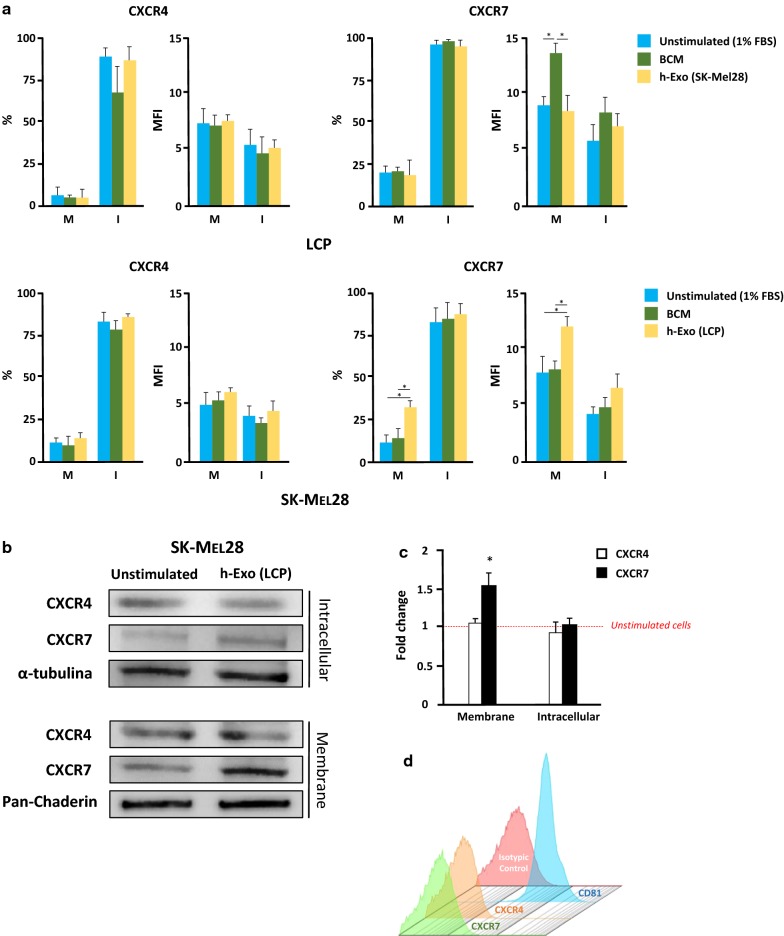
Expression of CXCR7 is enhanced by BCM in ‘osteotropic’ cells and is induced by h-Exos in ‘not-osteotropic’ cells. **a** Levels of CXCR4 and CXCR7 were measured by flow-cytometry in osteotropic LCP and not-osteotropic SK-Mel28 cells following the stimulation with BCM (green) or h-Exos (yellow). Unstimulated (1% FBS) cells were the controls (blue). Histograms are the percentage of positive cells and mean fluorescence intensity (MFI) ratio relative to membrane (M) and intracellular (I) expression of CXCR4 (left) and CXCR7 (right). Neither BCM, nor h-Exos influenced the expression of CXCR4 in LCP cells. However, the stimulation with BCM significantly improved the MFI ratio of membrane levels of CXCR7 in LCP, while no variation was induced by h-Exos. By contrast, the stimulation of SK-Mel28 with BCM failed to modify CXCR4 and CXCR7 levels, while both percentage of positive cells and MFI ratio relative to CXCR7 membrane expression were significantly up-regulated by LCP-derived h-Exos. **b**, **c** Western blots confirmed a significant up-regulation (≈ 1.5-fold increase; p < 0.05) of membrane CXCR7 induced by LCP-derived h-Exos, while intracellular expression was similar to baseline. **d** Image is representative of flow cytometry of LCP-derived Exos revealing the absence of CXCR4 and CXCR7 on Exo membranes. The same Exos stained for CD81 served as positive control. Results are expressed as fold change from basal mRNA concentration (copy/µl) and represent mean ± SEM. Bars represent mean ± SEM. *p < 0.05

To this purpose, we analyzed by flow-cytometry both membrane and intracellular levels of CXCR4 and CXCR7 in either stimulated or unstimulated cells. As shown in Table 1, LCP and both SK-Mel28 and WM-266 similarly expressed membrane (6.5%, 10.1% and 8.4%, respectively) and intracellular (88%, 84.6% and 87.1%) levels of CXCR4. By contrast, the levels of both membrane and intracellular CXCR7 by LCP (17.5% and 94.7%, respectively) were higher with respect to both SK-Mel28 (10.4% and 82.5%) and WM-266 cells (9.4% and 79.5%). As shown in Fig. 6a, following the stimulation with BCM, the percentage of LCP and SK-Mel28 expressing either membrane or intracellular CXCR4 remained unchanged with respect to unstimulated cells in a fashion almost similar to CXCR7. The stimulation of WM266 produced similar results. However, the MFI ratio relative to membrane CXCR7 by LCP cells significantly increased (13.6 ± 1.2 vs 8.3 ± 0.9; p < 0.05) after the stimulation with BCM, while remained unchanged in SK-Mel28 (7.6 ± 0.9 vs 7.5 ± 1.3) and WM-266 (data not shown). By contrast, the stimulation of SK-Mel28 cells with h-Exos from osteotropic LCP up-regulated the membrane levels of CXCR7 as compared to unstimulated cells (30.5% vs 10.4%; p < 0.01), while the intracellular expression was unaffected (87.1% vs 82.5%). In addition, the MFI ratio relative to membrane expression of CXCR7 was also enhanced in SK-Mel28 cells by the stimulation with h-Exos with respect to baseline (12.3 ± 1.0 vs 7.7 ± 1.8; p < 0.05). Similar results were observed for WM-266 cells stimulated with h-Exos from LCP (data not shown). Consistent with gene expression analyses, levels of CXCR4 by not-osteotropic cells were not influenced by LCP-derived Exos in a fashion similar to those of both CXCR4 and CXCR7 by LCP stimulated with h-Exos from SK-Mel28.

**Table 1:** Basal expression of chemokine receptors by melanoma cells

Cell line	CXCR4				CXCR7			
	Membrane		Intracellular		Membrane		Intracellular	
	%	MFI	%	MFI	%	MFI	%	MFI
LCP	6.5 ± 2.1	6.7 ± 1.4	88.0 ± 4.6	5.1 ± 2.0	***17.5***±***3.2***	8.6 ± 1.4	***94.7***±***4.9***	5.4 ± 1.7
SK-Mel28	10.1 ± 2.7	5.0 ± 0.9	84.6 ± 3.8	4.3 ± 1.7	10.4 ± 2.7	7.5 ± 0.7	82.5 ± 3.7	4.3 ± 1.1
WM-266	8.4 ± 1.8	5.6 ± 1.2	87.1 ± 4.2	4.5 ± 1.3	9.4 ± 2.5	6.8 ± 0.9	79.5 ± 4.2	4.1 ± 0.8
	*p 0.2*	*p 0.7*	*p 0.15*	*p 0.9*	*p 0.07*	*p 0.8*	*p 0.02*	*p 0.7*

Values are percentage of fluorescent cells and relative mean fluorescence intensity (MFI)

Membrane and intracellular levels of CXCR7 by LCP (bolditalics) were higher as compared to SK-Mel28 and WM-266 cells

Italic values refer to the *p* values calculated with “One-way ANOVA test” for the differences observed between cells in their CXCR4/CXCR7 basal levels

To verify these data, WB experiments were completed on the membrane and cytosolic fractions of SK-Mel28. As shown in Fig. 6b, c, the stimulation with h-Exos from LCP induced a significant up-regulation of membrane CXCR7 expression (1.53 ± 0.22-fold increase; p < 0.05), while the cytoplasmic fraction was apparently not influenced (1.06 ± 0.1-fold increase; p = ns). Additionally, no modification of CXCR4 expression was revealed in either membrane or cytoplasmic fractions of stimulated SK-Mel28 as compared to unstimulated cells (1.1 ± 0.07 and 0.88 ± 0.18-fold increase, respectively).

Levels of CXCR4 and CXCR7 were then measured by flow cytometry in Exos from osteotropic LCP. As shown in Fig. 6d, both chemokine receptors were not revealed in these Exos, thus excluding a direct Exo-mediated transfer of these molecules onto the plasma membrane of SK-Mel28 as a putative mechanism involved with CXCR7 up-regulation.

### Silencing of either CXCR4 or CXCR7 disables the osteotropism of melanoma cells

**Fig. 7:**
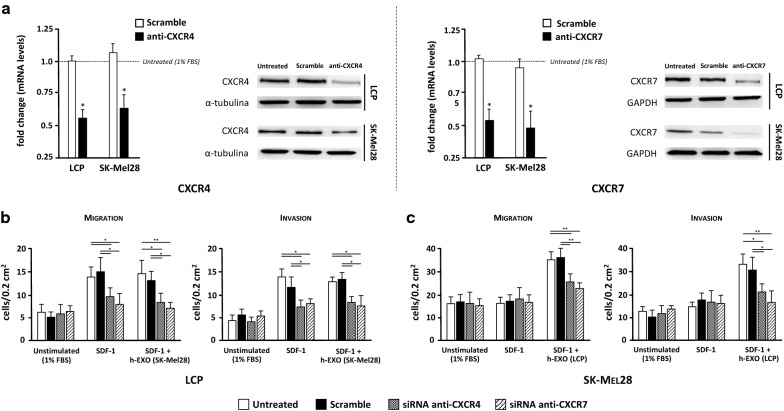
Small interfering RNAs of *CXCR4* and *CXCR7* disables the osteotropism of melanoma cells. The contribution of each chemokine receptor in the osteotropism of LCP and SK-Mel28 cells was investigated by trans-well assays after silencing of either *CXCR4* or *CXCR7* with dedicated siRNAs. **a** The transfection efficiency of anti-CXCR4 and anti-CXCR7 siRNAs was evaluated by dd-PCR (left) and WB (right). Treatment with siRNAs restrained more than 50% of mRNA levels of *CXCR4* and *CXCR7* in both cell lines with respect to untreated cells, while were unchanged in scramble-treated cells. A similar decrease in terms of protein expression was demonstrated in the same cell lines. Results are expressed as fold change from basal mRNA concentration (copy/µl). **b** LCP silenced for CXCR4 or CXCR7 were studied in terms of migratory (left) and invasive (right) capacity using SDF-1 as chemoattractant, either in presence or absence of h-Exos, and compared to both scramble and untreated cells. Melanoma cells not stimulated by SDF-1 were the negative controls. Both the migratory and invasive capacity of LCP were increased by SDF-1 stimulation in scramble and untreated cells as compared to unstimulated populations, although they were impaired by silencing of either CXCR4 or CXCR7. Similar results were obtained using the h-Exos from SK-Mel28 cells. **c** Migration (left) and invasion (right) of untreated, scramble and silenced SK-Mel28 cells were not influenced by SDF-1 with respect to unstimulated cells. By contrast, the stimulation with h-Exos from LCP increased the migration and invasion of untreated and scramble cells that, conversely, were dampened by treatment with anti-CXCR4 and anti-CXCR7 siRNAs. Bars are mean ± SEM. *p < 0.05; **p < 0.01

To investigate the functional relevance of chemokine receptors in the osteotropism of melanoma cells, silencing of either CXCR4 or CXCR7 were carried out by siRNAs. Efficient silencing of both mRNA and protein levels in LCP and SK-Mel28 cells was verified by both dd-PCR and WB. As shown in Fig. 7a, more than 50% of mRNA levels of CXCR4 and CXCR7 were restrained in both cell lines as compared with untreated cells. A similar decrease in terms of protein expression was also demonstrated. To avoid the possible contamination from other soluble factors released by bone fragments, human-recombinant SDF-1 was used as chemoattractant for migration and invasion assays (Fig. 7b, c). The effect of SDF-1 alone or in combination with h-Exos on CXCR4 and CXCR7 expression by LCP and SK-Mel28 cells was investigated by dd-PCR (Additional file 2: Figure S1) obtaining similar results to those observed by BCM stimulation. Similarly to previous experiments using bone fragments, migration of both untreated and scramble-treated LCP was significantly increased by SDF-1 as compared to unstimulated cells (13.7 ± 2.4 vs 6.1 ± 1.7 cells/0.2 mm^2^ and 14.9 ± 4.5 vs 5.2 ± 1.1 cells/0.2 mm^2^, respectively; p < 0.05) (Fig. 7b). However, the migration of cells stimulated with SDF-1 was dramatically impaired by siRNA of either CXCR4 (9.5 ± 1.9 cells/0.2 mm^2^) or CXCR7 (7.8 ± 2.4 cells/0.2 mm^2^) as compared to both untreated and scramble-treated cells (p < 0.05), while h-Exos were ineffective. Furthermore, both untreated and scramble-treated SK-Mel28 were not influenced by SDF-1 in their migratory property with respect to unstimulated cells (16.1 ± 3.5 vs 15.9 ± 2.8 cells/0.2 mm^2^ and 17 ± 3.7 vs 16.7 ± 3.2 cells/0.2 mm^2^, respectively; p = ns), and no effect was induced by SDF-1 in silenced cells (Fig. 7c). However, the concomitant stimulation of SK-Mel28 cells with SDF-1 and h-Exos from LCP significantly increased the migration of both untreated (33.6 ± 3.4 cells/0.2 mm^2^; p < 0.001) and scramble-treated cells (34.2 ± 4.1 cells/0.2 mm^2^; p < 0.001), while this effect was dampened in CXCR4 (25.4 ± 3.7 cells/0.2 mm^2^) and CXCR7 silenced cells (22.5 ± 2.8 cells/0.2 mm^2^). Similar results were obtained when LCP and SK-Mel28 cells were investigated for their invasive capacity under the same conditions.

These data proved that SDF-1 drives the chemotaxis of osteotropic melanoma cells through the CXCR4/CXCR7 axis that can be activated in not-osteotropic cells by Exos through the up-regulation of membrane CXCR7 molecules.

## Discussion

The events driving the metastatic organotropism of cancer cells depend on selective signaling interactions between cytokines, chemokines and relative receptors. The present study was aimed to explore the mechanisms regulating the bone tropism of melanoma cells and showed that, at least in vitro, Exos play a potential role in this process by reprogramming the osteotropism of these cells.

The machinery driving the colonization by melanoma cells of metastatic sites, including the bone, has been poorly investigated although previous studies correlated the CXCR3, CXCR4, CCR7 and CCR10 expression with the enhanced propensity to migrate to lymph nodes, lung and skin [22, 23]. Other findings, moreover, suggested that primary tumors are capable to spread molecular components to render the future metastatic organ suitable for the homing of detached cancer cells, thus early establishing a niche permissive for their survival and growth [4]. To this regard, many tumor-derived factors, such as the vascular endothelial growth factor (VEGF), tumor necrosis factor (TNF)-α, TGF-β, lysyl oxidase (LOX), versican and EVs have been reported to be potentially involved in the formation of the pre-metastatic niche [24]. In this context, Exos released by melanoma cells propagate pro-metastatic signals to distant recipient cells by delivering a cargo of active molecules that enhance the secretion of angiogenic factors, matrix metalloproteinases (MMPs) and immune-suppressive cytokines [25]. Here, we demonstrated that tumor-derived Exos enhance the in vitro chemotaxis of melanoma cells toward the SDF-1, thus suggesting a possible in vivo role of these EVs in bone metastasis formation.

The skeleton is the third most frequently affected metastatic site in cancer and its colonization occurs in 15% of patients with melanoma [1]. Concerning the molecular mechanisms, it has been suggested the role of osteopontin, a glycoprotein involved in cell adhesion and extracellular matrix remodeling, that regulates the migration of melanoma cells to the bone marrow niche [26, 27], while the high-expression of leukaemia inhibitory factor (LIF) has been recently associated with malignant osteolysis by the TGF-β pathway activation [28]. In this context, great regard has been reserved to tumor-derived Exos in relation to their ability to break-off the virtuous cycle of bone remodeling [29]. In fact, uptake of tumor-derived Exos by bone marrow, endothelial and mesenchymal stem cells (MSCs) has been proven and the integrins expressed by Exos apparently play a role in driving their fusion with these target cells [30–33]. Furthermore, Exos influence the expression of chemokine receptors in recipient cancer cells and reprogram their EMT machinery [34, 35].

This aspect has been explored in the present research and here we provide evidence that Exos from osteotropic melanoma cells induce, at least in vitro, chemoattraction to the bone in not-osteotropic cells. To identify the pivotal events enrolled in these properties, we first investigated the baseline mRNA levels of genes involved in EMT, chemotaxis and bone metastasis development. These genes were almost similarly expressed by the osteotropic and not-osteotropic melanoma models. However, a slight up-regulation of genes implicated in EMT was revealed, while the modest increase of *N*-*CAD*, *ZEB1*, *MMP1* and *MMP2* in not-osteotropic cells was probably correlated to their high migratory and invasive properties. On the other hand, we explored a limited set of genes potentially involved in bone tropism and it is possible that our cellular models are not constitutively activated in osteotropism. Therefore, we reasoned that osteotropic and not-osteotropic cells could be differently activated to this function by the stimulation of soluble factors mostly produced by bone fragments. This was further confirmed since osteotropic LCP stimulated by BCM up-regulated both *CXCR4* and *CXCR7* as well as *MMP1, PTH*-*rP* and *TGF*-*β*, while the gene profile was unchanged in not-osteotropic SK-Mel28 and WM-266.

Although the EMT process is a major determinant of melanoma metastasis leading cancer cells to detach from the primary tumor and spread toward distant sites throughout the bloodstream [36], our data suggest that further events in response to stimuli from bone microenvironment are required to activate their homing to the bone niche. This model confirms previous studies revealing that the exposure of cancer cells to osteoblast conditioned medium can increase the expression of dysadherin and CCL2, thus positively influencing their migratory properties [37]. However, it is conceivable that only those cells endowed with an innate osteotropic behaviour can be activated by the signals from bone marrow accessory cells and thus, a minority of melanoma cell sub-populations are able to originate bone metastases. In this complex context, although their role in malignant osteoclastogenesis is unclear, Exos may participate to the bone metastatic process favoring the interplay among heterogeneous cancer cells in the tumor microenvironment. Here, we investigated Exos in influencing the in vitro osteotropism of melanoma cells and found that they enhance their attraction to bone fragments. Gene and protein expression profile of melanoma cells showed the up-regulation of CXCR7 as a mechanism apparently critical for this process. Thus, it is possible that osteotropic melanoma cells reprogram the phenotype of not-osteotropic cells through CXCR7 up-regulation induced by exosomal transfer of active cargos resulting in activation of the chemotactic machinery of migration/invasion toward signals originated within the bone marrow niche.

Both CXCR7 and CXCR4 are seven-transmembrane G-protein coupled receptors with selective binding with SDF-1, whose affinity for CXCR7 is tenfold higher than for CXCR4 [38]. Despite SDF-1-driven intracellular signals via CXCR4 have been widely demonstrated in the regulation of cancer cell homing to the bone [39], the role of CXCR7 is still debated. The absence of a typical G-protein-mediated response following CXCR7 binding to SDF-1 suggested a role as a modulator of CXCR4 signaling, thus acting as decoy receptor or scavenger for SDF-1 sequestration [40]. However, it has been recently demonstrated that CXCR7 triggers the MAPK cascade via β-arrestins2/Erk1 [41], whose activation is implicated in the migration of melanoma cells [42]. Data from our functional experiments using anti-CXCR7 siRNAs suggest that tumor-derived Exos reprogram in vitro the osteotropic behaviour of melanoma cells induced by SDF-1 through membrane CXCR7 up-regulation. In addition, by silencing the CXCR4 receptor we also demonstrated that CXCR4 and CXCR7 co-expression by melanoma cells drive their SDF-1-mediated chemotaxis independently from Exo stimulation. In this context, CXCR7 is apparently necessary to hold melanoma cells susceptible to SDF-1/CXCR4 signaling by working as SDF-1 neutralizer, thus keeping its gradient functionally active nearby the cell surface [43–45]. Consistent with this hypothesis, we observed an increased expression of membrane CXCR7 in osteotropic LCP cells following their stimulation with BCM while a similar enrichment was demonstrated by stimulating not-osteotropic SK-Mel28 and WM-266 cells with LCP-derived Exos. Since Exos from LCP were largely clustered in the cytoplasm of recipient not-osteotropic cells and resulted negative for CXCR4 and CXCR7 expression, we reasoned that membrane CXCR7 up-regulation was related to a defined gene activation program rather than to a protein transfer. These findings were also strengthened by siRNA experiments.

## Conclusions

**Fig. 8:**
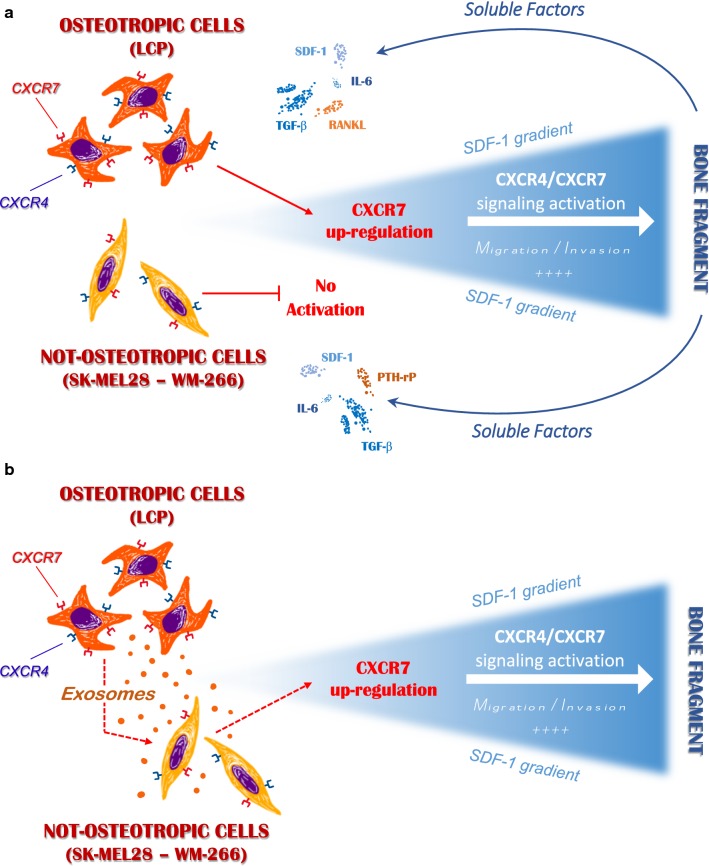
Possible role of melanoma-derived Exos in regulating the in vitro osteotropism of melanoma cells. **a** Soluble factors released by the bone fragment up-regulate membrane-CXCR7 levels in osteotropic melanoma cells (LCP), thus stimulating their migration towards the SDF-1 through the CXCR4/CXCR7 signaling activation. By contrast, not-osteotropic cells (SK-Mel28 and WM-266) are refractory to the stimulation from bone-derived soluble factors and SDF-1-mediated chemoattraction cannot be activated. **b** Exosomes released by osteotropic melanoma cells stimulate not-osteotropic cells to increase membrane levels of CXCR7 resulting in a reprogramming effect on their original chemoattraction toward SDF-1

In our in vitro model, melanoma cells are endowed with different bone tropism, mostly depending on SDF-1/CXCR4/CXCR7 axis activation in response to bone microenvironment stimulation. As represented in Fig. 8, we suggest that innate propensity of melanoma cells to migrate and invade toward the SDF-1 gradient depends on the up-regulation of membrane CXCR7 following their exposition to bone-derived soluble factors. Notwithstanding the mechanisms driving CXCR7 rearrangements under Exo stimulation have not been investigated in the present research, extensive proteomics and RNA profiling of melanoma-derived Exos have previously identified transcription factors and miRNAs that regulate cell adhesion, motility and invasiveness [46, 47]. Therefore, additional efforts are required to define those factors putatively implicated in the CXCR7 modulation that are produced by bone accessory cells or delivered by Exos. Thus, definite in vivo studies to address these findings are needed for the identification of alternative strategies and druggable targets for the treatment and prevention of melanoma bone metastasis.

## Additional files

**Additional file 1: Table S1.** Primer sequences used for qRT-PCR and dd-PCR experiments.

**Additional file 2: Figure S2.** Effects of SDF-1 stimulation on *CXCR4/CXCR7* expression. LCP and SK-Mel28 cells were analyzed by dd-PCR to investigate the effects of 6-h stimulation with recombinant SDF-1 (100 ng/ml) and h-Exos (50 µg/ml) on *CXCR4*/*CXCR7* expression. The stimulation of osteotropic LCP with SDF-1 (white bars) produced a significant increase of both *CXCR4* (2.03 ± 0.4-fold change) and *CXCR7* (3.4 ± 0.5-fold change) mRNA levels. Similar results were obtained with the addition of h-Exo (black filled bars) from SK-Mel28 cells. On the other hand, *CXCR4* and *CXCR7* levels were mostly unchanged following stimulation of not-osteotropic SK-Mel28 cells with SDF-1 (0.93 ± 0.1 and 1.27 ± 0.3 fold change, respectively), while *CXCR7* only resulted significantly increased (3.5 ± 0.2-fold change) in the presence of SDF-1 and h-Exos from osteotropic LCP. Bars are mean ± SEM. *p < 0.05; **p < 0.01; ***p < 0.001.

## Data Availability

All data generated or analyzed during this study are included in this published article and its Additional files.

## Abbreviations

a-Exos: autologous exosomes
h-Exos: heterologous exosomes
BCM: bone conditioned medium
BSA: bovine serum albumin
CANX: calnexin
CAD: cadherin
CCL: C-C chemokine ligand
CCR: C-C chemokine receptor
CXCR: C-X-C chemokine receptor
dd-PCR: droplet digital-PCR
EMT: epithelial-to-mesenchymal transition
EVs: extracellular vesicles
FBS: fetal bovine serum
IL: interleukin
LIF: leukaemia inhibitory factor
LOX: lysyl oxidase
miRNAs: microRNAs
MFI: mean fluorescence intensity
MMPs: matrix metalloproteinases
MoAb: monoclonal antibodies
MSCs: mesenchymal stem cells
PTHrP: parathyroid hormone-related peptide
RANKL: nuclear factor kappa-B ligand
RT-PCR: real time-PCR
SDF: stromal derived factor
siRNAs: small interfering-RNAs
SRE: skeletal-related events
TEM: transmission electron microscopy
TGF: transforming growth factor
TNF: tumor necrosis factor
VEGF: vascular endothelial growth factor
WB: Western blot
