# Supplementary material for: Tumor-derived exosomes promote the in vitro osteotropism of melanoma cells by activating the SDF-1/CXCR4/CXCR7 axis
Source: J Transl Med. 2019 Jul 19;17:230. doi: 10.1186/s12967-019-1982-4 (PMC6642540; doi:10.1186/s12967-019-1982-4)
Supplement: Supplementary file 1 — Additional file 1: Table S1. Primer sequences used for qRT-PCR and dd-PCR experiments. [file 12967_2019_1982_MOESM1_ESM.docx]

| *Gene* | *Forward primer* | | *Reverse primer* |  |
| --- | --- | --- | --- | --- |
| *CCR6*  *CCR7*  *CCR10*  *CDH1*  *CDH2*  *CDH13*  *CXCR1*  *CXCR2*  *CXCR4*  *CXCR7*  *ILK*  *MMP1*  *NOTCH3*  *PDGFRα*  *PIK3A*  *PTHrP*  *SMAD2*  *SMAD3*  *SNAIL2*  *TGFβ1*  *TWIST1*  *ZEB1*  *ZEB2* | 5’-TGAAGGACCTGTGGTGTGTG-3’  5’-GGTGTACAGGACCAAGAGG-3’  5’-TGCTGCTGGATACTGCCGAT-3’  5’-AGCAGAACTAACACACGGGG-3’  5’-CCACTCCAACTGGTATCTT-3’  5’-GGCAATTGACAGTGGCAACC3’  5’-TGGCCGGTGCTTCAGTTAG-3’  5’-CCCAGGTCAGAAGTTTCATCGT-3’  5’-TGGGTGGTTGTGTTCCAGTT-3’  5’-GGCCTGTTCTCAATCTGGTCT-3’  5’-TGTCGACTGCTACCACGG-3’  5’-AGCTAGCTCAGGATGACATTGATG-3’  5’-CCTGTGGCCCTCATGGTATC-3’  5’-CGCGGTTTTTGAGCCCATTA-3’  5’-CCCCGAGCGTTTCTGCTTTG-3’  5’-GGATGATGCTCCAAAGGGGA-3’  5’-AAAGGGTGGGGAGCAGAATAC3’  5’-AGTTGAGGCGAAGTTTGGGC-3’  5’-CTTTTCTTGCCCTCACTGC-3’  5’-CGCGTGCTAATGGTGGAAAC-3’  5’-CCGGAGACCTAGATGTCATTGT-3’  5’-CAGGCGAGCAGTGTGACT-3’  5’-CAGAAGCCACGATCCAGACC-3 | 5’-AGGAGACGCATTGTCGTTA-3’  5’-AGGATCAGCATGTCCGGTTC-3’  5’-GGCGTAGAGAACGGGATTGA-3’  5’-ACCCACCTCTAAGGCCAT-3’  5’-TCTACTGCATGTGCCCCTAA-3’  5’-TGCAGGAGCACACTTGTACC-3’  5’-GGAACACTAGGGCATAGGCG-3’  5’-AGCAGCTGTGACCTGCTGTTA-3’  5’-TTGGAGTGTGACAGCTTGGA-3’  5’-TTCAAGTTCCCAGACCCCAC-3’  5’-GAGGACTGTGGAGTGATCCAG-3’  5’-GCCGATGGGCTGGACAG-3’  5’-CATGGGTTGGGGTCACAGTC-3’  5’-GCTCTGGGAAACTTCTCCTCC-3’  5’-TTCTTGGGGGCATCAAGTGG-3’  5’-TTTCTAGTGCCACTGCCCAT-3’  5’-CTCCAGAATATGCAAGAATGCAATG-3’  5’-GAAAGGCAGGATGGACGACA-3’  5’-ACAGCAGCCAGATTCCTCAT-3’  5’-GTTCAGGTACCGCTTCTCGG-3’  5’-CCCACGCCCTGTTTCTTTGA-3’  5’-GCAAGACCGACGACCTGAT-3’  5’-GTCACTGCGCTGAGGTACT-3 | | |

**Table S1:** Primer sequences used for qRT-PCR and dd-PCR experiments
