# Supplementary figures and images for: Tumor-derived exosomes promote the in vitro osteotropism of melanoma cells by activating the SDF-1/CXCR4/CXCR7 axis
Source: J Transl Med. 2019 Jul 19;17:230. doi: 10.1186/s12967-019-1982-4 (PMC6642540; doi:10.1186/s12967-019-1982-4)

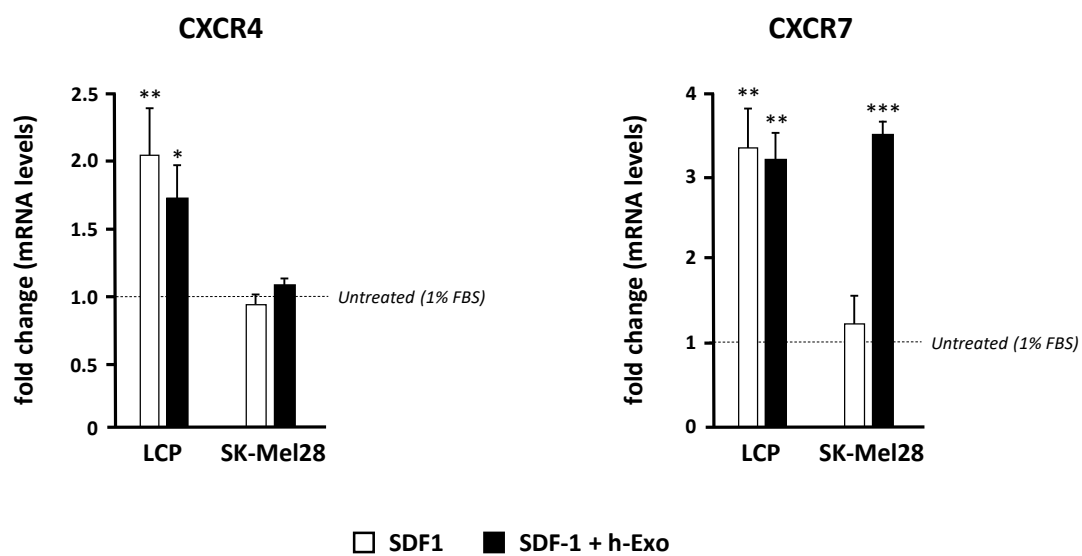

Figure S1

Supplement: Supplementary file 2 — Additional file 2: Figure S2. Effects of SDF-1 stimulation on CXCR4/CXCR7 expression. LCP and SK-Mel28 cells were analyzed by dd-PCR to investigate the effects of 6-h stimulation with recombinant SDF-1 (100 ng/ml) and h-Exos (50 µg/ml) on CXCR4/CXCR7 expression. The stimulation of osteotropic LCP with SDF-1 (white bars) produced a significant increase of both CXCR4 (2.03 ± 0.4-fold change) and CXCR7 (3.4 ± 0.5-fold change) mRNA levels. Similar results were obtained with the addition of h-Exo (black filled bars) from SK-Mel28 cells. On the other hand, CXCR4 and CXCR7 levels were mostly unchanged following stimulation of not-osteotropic SK-Mel28 cells with SDF-1 (0.93 ± 0.1 and 1.27 ± 0.3 fold change, respectively), while CXCR7 only resulted significantly increased (3.5 ± 0.2-fold change) in the presence of SDF-1 and h-Exos from osteotropic LCP. Bars are mean ± SEM. *p < 0.05; **p < 0.01; ***p < 0.001. [file 12967_2019_1982_MOESM2_ESM.pdf]
